# Bridging tradition and modernity: mitochondrial dynamics as a Traditional Chinese Medicine therapeutic target in cardiovascular disease

**DOI:** 10.1186/s13020-025-01225-8

**Published:** 2026-02-09

**Authors:** Chengyu Du, You Yu, Meng Li, Jin Zhou, You Wang, Xuefeng Guo, Huan Zhang, Zheng Li, Yelei Han, Min Pang, Rui Yu

**Affiliations:** 1 Liaoning University of Chinese Medicine, Shenyang, 110032 China; 2 Liaoning University of Traditional Chinese Medicine Affiliated Second Hospital, Shenyang, 110034 China

**Keywords:** Mitochondrial dynamics, Traditional Chinese medicine, Cardiovascular disease, Multi-target therapy, TCM and mitochondrial dynamics

## Abstract

**Background:**

A pathological link exists between mitochondrial fission/fusion imbalance and cardiovascular disease (CVD). Traditional Chinese Medicine (TCM), based on concepts such as "Qi stagnation and blood stasis" and "Yin-Yang imbalance," helps balance mitochondrial function through the combined effects of multiple components, providing a comprehensive treatment approach for CVD.

**Aims of the study:**

To methodically clarify the molecular processes by which TCM formulations, extracts, and bioactive compounds target mitochondrial dynamics to intervene in CVD over the past five years, highlighting their ethnopharmacological significance in "multi-component and multi-target" synergistic actions.

**Methods:**

This study searched PubMed, Web of Science, Wanfang, and VIP databases (2019–2024), using the Boolean search formula: ("cardiovascular disease" OR "CVD") AND ("mitochondrial dynamics" OR "mitochondrial fission" OR "mitochondrial fusion") AND ("Traditional Chinese Medicine" OR "TCM") AND ("active compounds" OR "bioactive components"). After deduplication with EndNote, 183 articles were systematically screened and included, comprising in vitro experiments using cardiomyocyte models, in vivo studies based on animal models of CVD, and mechanistic investigations utilizing ex vivo tissues or cellular experiments (all human clinical trials were excluded).

**Results:**

Formulations such as Buyang Huanwu Decoction (BYHWD) and Qishen Yiqi Dropping Pills (QSYQ) improved heart conditions by reducing dynamin-related protein 1 (Drp1) overactivity and increasing mitofusin 2 (Mfn2) and optic atrophy 1 (OPA1) levels. Bioactive compounds, such as salidroside(Sal), prevented Drp1 from causing mitochondria to split apart by activating the AMP-activated protein kinase(AMPK)/Sirtuin 1(SIRT1) pathway, while astragaloside IV facilitated better mitochondrial fusion to enhance energy utilization.

**Conclusion:**

TCM manages mitochondria dynamics through multi-target mechanisms, connecting "overall treatment" with "specific targeting" for heart disease therapy. Further ethnopharmacological translation requires standardized screening of bioactive components and the development of innovative drug delivery systems. The study suggests a "Traditional Chinese Medicine-Mitochondrial Dynamics Intervention Model (TCM-MDIM)," which combines organelle-level mitochondrial regulation with the principle of balancing blood and qi to offer novel approaches to the targeted therapy of cardiovascular disorders.

**Graphical abstract:**

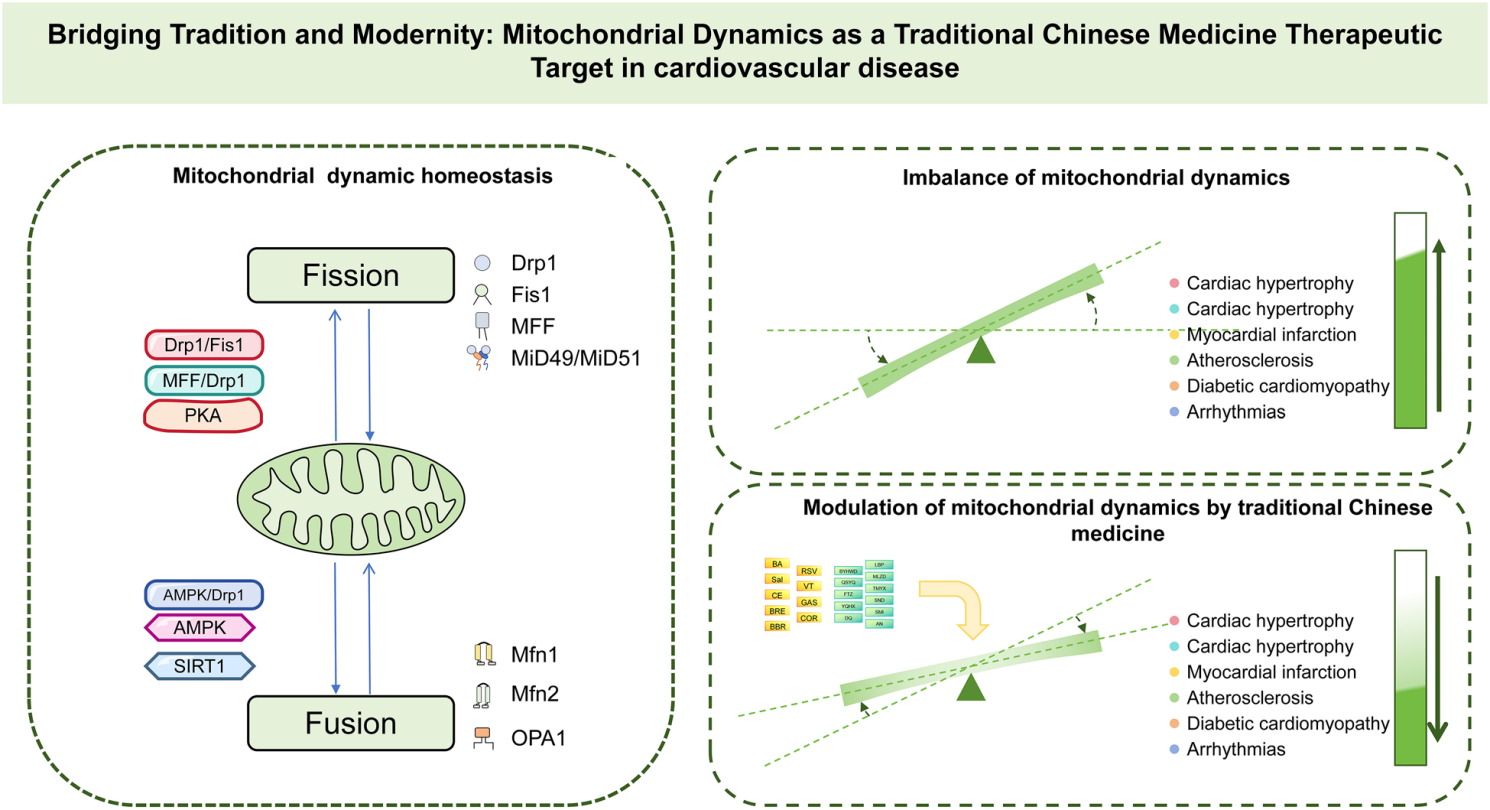

## Introduction

Globally, cardiovascular disease (CVD) remain one of the leading causes of morbidity and mortality worldwide [1–4]. Mounting evidence indicates that mitochondrial dysfunction, particularly imbalance in mitochondrial dynamics, plays a pivotal role in the pathogenesis of CVD [5]. By triggering excessive generation of reactive oxygen species (ROS), disruption of calcium homeostasis, and energy metabolism disorders [6, 7], it ultimately leads to cardiomyocyte death. While beta-blockers and angiotensin-converting enzyme inhibitors (ACEIs) are classic drugs in the cardiovascular field, their single-target mechanisms struggle to simultaneously address the complex multi-pathological aspects of CVD. Their clinical application is limited by severe adverse reactions (e.g., beta-blockers may induce asthma or exacerbate heart failure (HF) [8], while ACEIs are prone to causing dry cough [9, 10]). These limitations make it difficult to meet the demands of complex pathological conditions, highlighting the urgent need to develop novel multi-target synergistic therapeutic strategies. In contrast, Traditional Chinese Medicine (TCM), with its holistic approach and "multi-component, multi-target" therapeutic strategy, holds promise for achieving more synergistic protective effects by simultaneously regulating multiple pathological pathways such as mitochondrial dynamics (e.g., fission/fusion), ROS production, calcium homeostasis, and energy metabolism. This offers a novel perspective for overcoming the limitations of single-target western drug therapies.

Rooted in the holistic medical philosophy of ancient China, TCM employs "pattern differentiation-based therapy (Bianzheng Lunzhi)" to prevent and treat cardiovascular issues, emphasizing the harmonization of Qi-Blood and Yin-Yang equilibrium as core therapeutic principles [11, 12]. Modern pharmacological studies have validated the cardioprotective effects of multiple compounds derived from TCM. Emerging evidence suggests that TCM exerts therapeutic benefits by modulating mitochondrial dynamics, targeting key regulators such as dynamin-related protein 1 (Drp1), mitofusins 1 and 2 (Mfn1/2), and optic atrophy 1 (OPA1). For instance, Xuanfei Baidu Decoction—a classical herbal formula—and nodakenin (a major coumarin derivative from Angelica sinensis) mitigate myocardial injury by regulating Drp1-mediated mitochondrial fission [13, 14]. In recent years, the application of high-throughput omics technologies for analyzing active components of TCM and the use of organoid models are driving the transformation of our understanding of TCM's regulatory mechanisms on mitochondrial dynamics from "empirical description" to "precise elucidation". Unlike previous reviews that focused on single-pathway regulation, this study is the first to systematically elucidate how TCM compound formulas, preparations, crude extracts, and broad-spectrum active monomers​​ achieve "multi-component, multi-target" dynamic balance by synergistically regulating mitochondrial fission proteins (Drp1 phosphorylation) and fusion proteins (Mfn2/OPA1 expression). It translates the TCM theories of "harmonizing qi and blood" and "yin-yang balance" into verifiable organelle-level regulatory mechanisms. This strategy provides an innovative solution for the staged management of cardiovascular diseases, integrating traditional wisdom with modern scientific approaches.

This study systematically elucidates the molecular mechanisms by which TCM has targeted mitochondrial dynamics to intervene in CVD over the past five years, adopting a systematic framework of "molecular mechanisms—core pathways—disease pathology—TCM interventions": First, it elucidates the molecular mechanisms of mitochondrial fission/fusion dynamics (e.g., the Drp1/Mfn2/OPA1 axis); then, it analyzes key regulatory pathways (e.g., AMP-activated protein kinase(AMPK)/Sirtuin 1(SIRT1) etc.); subsequently, it focuses on the pathological processes of different CVD types (e.g., myocardial ischemia/reperfusion injury (MIRI), atherosclerosis (AS), etc.); and finally, it systematically categorizes TCM intervention strategies (compound formulas/preparations/crude extracts/broad-spectrum active monomers) and their multi-target synergistic effects, clarifying the ethnopharmacological value of TCM in integrating "holistic regulation" with "precise intervention". Additionally, we discuss the challenges in translating basic research findings into clinical practice and propose future directions for the development of integrated medicine. To achieve a mechanism-driven transformation of conventional medical wisdom, this study develops the "Traditional Chinese Medicine-Mitochondrial Dynamics Intervention Model (TCM-MDIM)" framework, which links molecular targets of mitochondrial dynamics (Drp1/Mfn2/OPA1 axis) with holistic regulation in TCM (qi/blood, yin/yang balance).

## Integrated mechanisms of mitochondrial dynamics in CVD

### Molecular mechanisms of mitochondrial dynamics

**Fig. 1:**
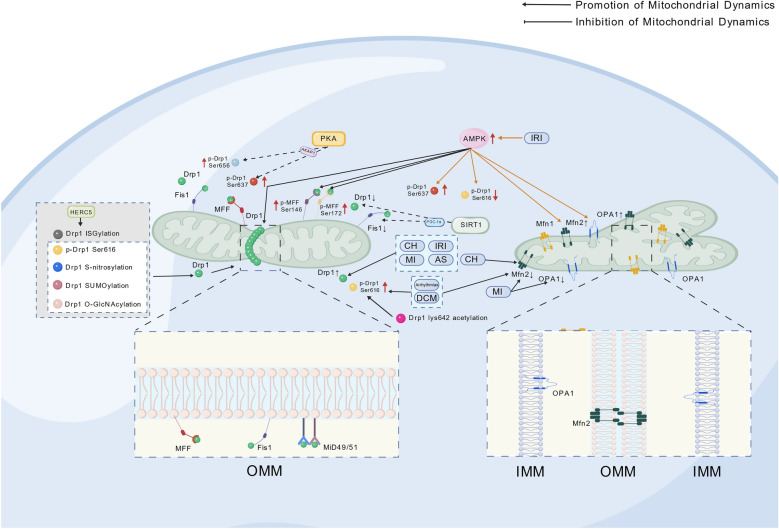
Integrated regulatory mechanisms of mitochondrial dynamics in CVD (all figures created with BioGDP.com [46]). The schematic diagram illustrates the molecular mechanisms of mitochondrial fission and fusion processes and their core regulatory pathways. The left panel displays the fission process: Drp1 is recruited to the OMM via adaptor proteins (Fis1, MFF, MiD49/51) and completes membrane scission through GTP hydrolysis; its activity is regulated by various post-translational modifications, such as Ser616/Ser637 phosphorylation, SUMOylation, and S-nitrosylation. The right panel shows the fusion process: Mfn1/2 mediates outer membrane fusion, while OPA1 (long and short forms) drives IMM fusion dependent on membrane potential and the lipid microenvironment. Central signaling hubs (e.g., PKA, AMPK, SIRT1-PGC-1α) dynamically balance fission and fusion by modifying Drp1, Mfn2, and OPA1, participating in the regulation of mitochondrial network stability, energy metabolism, and cell fate in CVD. Arrows indicate the direction of regulation, and colors distinguish different molecular categories

Mitochondrial dynamics maintain cellular energy homeostasis through a precise balance of fission and fusion, with the core regulatory proteins Drp1 and OPA1 governing mitochondrial division and integration, respectively [15, 16]. The fission process begins with the recruitment of Drp1 from the cytoplasm to the outer mitochondrial membrane (OMM), mediated cooperatively by adapter proteins such as Mitochondrial fission 1 protein (Fis1), Mitochondrial dynamics proteins of 49 and 51 kDa (​MiD49/51), and Mitochondrial fission factor(MFF) [17, 18]. Subsequently, Drp1 oligomerizes into a spiral structure and completes membrane scission via GTP hydrolysis [6, 19–21]. This process is dynamically and finely regulated by post-translational modifications. Modifications at different sites precisely control Drp1 recruitment to mitochondria by altering its conformation and affinity for OMM adapter proteins (e.g., Fis1, MFF, MiD49/51): phosphorylation at Ser616 enhances its binding to the OMM and oligomerization [22], while phosphorylation at Ser637 is activated by Protein kinase A(PKA) and dephosphorylated by calcineurin (CaN) to inhibit fission [23, 24]. SUMOylation stabilizes Drp1 binding to the OMM [25], S-nitrosylation promotes Drp1 oligomerization and GTPase activity [26], ISGylation mediated by HERC5 facilitates mitochondrial fission [27], O-GlcNAcylation increases the GTP-bound active form of Drp1 and induces its translocation to mitochondria [28], and acetylation of Drp1 at K642 promotes its Ser616 phosphorylation, enhances oligomerization and GTPase activity, driving its translocation to the OMM and interaction with voltage-dependent anion channel 1 (VDAC1) [29], a process reversible by Sirt1 activators [30]. The fusion mechanism occurs in two steps: Mfn1/2 first anchors adjacent OMMs and drives GTP-dependent membrane fusion [16, 20, 31], followed by Long-form optic atrophy 1 (L-OPA1) initiating inner membrane fusion pore formation via trans-cardiolipin interactions. Short-form optic atrophy 1 (S-OPA1) can bind to liposomes and induce local membrane curvature to promote the stabilization and expansion of inner membrane (IMM) fusion pores [32], a process requiring synergistic action of membrane potential and lipid microenvironment (in Fig. 1 and Table 1).

### Signaling hubs of mitochondrial dynamics: molecular targets for CVD therapy

The precise regulation of mitochondrial fission and fusion relies on the synergistic action of core signaling hubs. The AMPK/Drp1 axis functions as an energy-sensing center, recruiting Drp1 to mitochondria via phosphorylation of MFF (Ser172/Ser146) [35, 36]. In diabetic myocardial microvascular disease, AMPK activation bidirectionally regulates Drp1 phosphorylation (inhibiting Ser616/promoting Ser637) to suppress pathological fission [36–40], while simultaneously enhancing fusion capacity by stabilizing Mfn2/OPA1 [40–43], demonstrating its metabolic context-dependent regulatory characteristics. The SIRT1–peroxisome proliferator-activated receptor gamma coactivator 1-alpha (PGC-1α) pathway acts as an NAD⁺-dependent regulatory module. In a low-pressure hypoxic heart model, upregulation of SIRT1 inhibits Drp1 and Fis1 expression [44], while dexmedetomidine inhibits Drp1 (Ser616/Ser637) phosphorylation and promotes OPA1-mediated fusion by activating SIRT1/PGC-1α [45], confirming its central role in endogenous protection. Notably, the SIRT1–PGC-1α pathway is not only a crucial regulator of mitochondrial dynamics balance but also a key bridge connecting dynamics to mitophagy. Activation of this pathway significantly enhances PTEN-induced kinase 1 (PINK1)/Parkin RBR E3 ubiquitin-protein ligase (Parkin)-mediated mitophagy levels, indicating its role as a pivotal hub in maintaining mitochondrial quality control [46]. Similarly, another study showed that SIRT1/PGC-1 pathway activation can induce mitophagy, thereby alleviating oxidative damage in intestinal epithelial cells [47]. In summary, the SIRT1–PGC-1α pathway plays a vital integrative regulatory role in cellular metabolic homeostasis by coordinating mitochondrial fusion and mitophagy processes. A-kinase anchoring protein 1 (AKAP1) promotes excessive mitochondrial fission in specific cell types such as podocytes by recruiting protein kinase A (PKA) to the mitochondrial surface, directly catalyzing phosphorylation of Drp1 at Ser637 [48]. Furthermore, the PKA-Drp1 network exhibits cell type specificity: In neurons, PKA/AKAP1-mediated phosphorylation of Drp1 Ser656 elongates mitochondria and maintains metabolic homeostasis [49], but silica nanoparticles induce cardiomyocyte apoptosis by activating the PKA-Drp1 axis [50], revealing its pathological role in cardiovascular toxicity (in Fig. 1 and Table 2).

### Characteristics of mitochondrial dynamic imbalance in CVD

The occurrence and progression of CVD are accompanied by characteristic mitochondrial dynamic imbalances: In cardiac hypertrophy (CH), pressure overload induces significant suppression of Mfn2 expression and Drp1 overexpression, triggering excessive mitochondrial fission and energy metabolism disorders [21, 50–54]. MIRI manifests as hypoxia-induced increased mitochondrial translocation of Drp1, while dysfunction of fusion proteins further amplifies mitochondrial network disruption. For example, formin Diaphanous-1 (DIAPH1)-Mfn2 complex affects cytoskeletal structure through its F-actin polymerization activity, thereby altering the relative positions of the endoplasmic reticulum (ER) and mitochondria, increasing the risk of infarct expansion [54–59]. Notably, the regulatory direction of AMPK exhibits energy stress level dependency: In cerebral ischemia models, moderate AMPK activation promotes mitochondrial fission and mitophagy by enhancing Drp1-Ser637 phosphorylation, clearing damaged mitochondria (in Fig. 1); whereas in MIRI models, high-intensity AMPK activation inhibits pathological mitochondrial fission by suppressing Drp1-Ser616 phosphorylation, enhancing Drp1-Ser637 phosphorylation, and upregulating Mfn1/Mfn2 expression [40, 59]. The core pathology of myocardial infarction (MI) is the significant downregulation of Mfn2/OPA1 and abnormal upregulation of the fission key factor Drp1 after coronary occlusion, leading to excessive mitochondrial fragmentation and proteasome overactivation specifically degrading Mfn2 [60–63]. Simultaneously, Drp1-mediated excessive fission amplifies cardiomyocyte death by inducing mitochondrial membrane potential collapse and pro-apoptotic factor release. Targeting these mechanisms, intervention strategies have shown significant efficacy: The fission inhibitor Mdivi-1 combined with fusion promoters reverses mitochondrial fragmentation and restores cognitive and cardiac function in MI rats [64], while inhibiting proteasome activity maintains mitochondrial structure and function by stabilizing Mfn2 levels [65]. A typical hallmark of AS is Drp1-mediated excessive mitochondrial fission, which promotes M1 polarization via the mitochondrial reactive oxygen species (mtROS)/NOD-like receptor thermal protein domain-associated protein 3 (NLRP3) inflammasome axis, accelerating plaque progression [65–71]. Diabetic cardiomyopathy (DCM) presents with suppressed Mfn2 expression and abnormal phosphorylation of Drp1 Ser616, leading to uncoupled energy metabolism [71–75]. The pathological mechanism of arrhythmia involves a multi-dimensional regulatory network. Its core link begins with ryanodine receptor 2 (RyR2) overactivation causing sarcoplasmic reticulum (SR) calcium leakage [76]. Subsequently, ​​RYR2-mediated calcium leakage increases mitochondrial calcium uptake via the mitochondrial calcium uniporter (MCU), activating Ca^2^⁺/calmodulin-dependent kinase II (CaMKII), which directly phosphorylates Drp1 at Ser616, thereby promoting Drp1 activation and mitochondrial fission [77]. Excessive CaMKII activation further exacerbates Drp1 Ser616 phosphorylation [78], collectively leading to energy metabolism disorders and apoptosis. This process triggers a mitochondrial ROS burst, forming a self-amplifying pro-arrhythmic positive feedback loop [79]. Additionally, this mechanism is characterized by Mfn2 deficiency disrupting mitochondrial-sarcoplasmic reticulum structural coupling, while excessive phosphorylation of Drp1 Ser616 ultimately causes calcium cycling disorders and disrupts mitochondrial dynamics balance [80, 80] (in Fig. 1).

## TCM targeting mitochondrial dynamics for CVD treatment

Mitochondrial dynamic imbalance is a key pathological mechanism of CVD progression, involving energy metabolism dysregulation, oxidative stress exacerbation, and apoptotic signaling activation. Leveraging TCM’s holistic philosophy and multi-target regulatory properties, numerous compound formulas and bioactive monomers have shown efficacy in improving CVD by modulating mitochondrial dynamics networks. This section systematically reviews representative TCM compound formulas, preparations, crude extracts and broad-spectrum active monomers with their molecular synergies.

### TCM compound formulas

#### Buyang Huanwu decoction (BYHWD)

Buyang Huanwu Decoction(BYHWD), a classical TCM formula, comprises seven herbs: *Astragalus membranaceus*(Huang Qi), *Angelica sinensis* (Oliv.) *Diels*(Dang Gui), *Paeonia lactiflora Pall.*(Chi Shao), *Conioselinum acuminatum* (Franch.) *Lavrova*(Chuan Xiong), *Prunus persica* (L.) *Batsch*(Tao Ren), *Carthamus tinctorius L.*(Hong Hua), and *Pheretima aspergillum (E. Perrier)* (Di Long). It is conventionally employed to stimulate Qi, enhance blood circulation, and alleviate stasis [82, 83]. Originally prescribed for post-stroke sequelae, recent studies extend its applications to cerebrovascular and CVD, including cerebral ischemia, AS, and myocardial injury [83–87]. Its multi-target mechanisms encompass anti-inflammatory, antioxidant, angiogenic, and tissue-repair effects, with emerging research focusing on mitochondrial dynamics regulation, offering novel insights for CVD therapy. In CVD pathology, mitochondrial dynamic imbalance (excessive fission/fusion) drives endothelial dysfunction, cardiomyocyte apoptosis, and fibrosis. In streptozotocin (STZ)-induced diabetic mice, BYHWD suppresses mitochondrial hyperfission by targeting the AMPK-Drp1 axis. Core components Astragalus membranaceus and PALCPP downregulate Drp1 expression, reduce mtROS production, and improve endothelial function to delay plaque progression [88]. In summary, BYHWD orchestrates multi-component synergies to precisely regulate fission/fusion equilibrium and redox homeostasis, intervening in CVD pathology at the organelle level. This "formula-multi-target "paradigm offers integrated strategies for CVD management and highlights the potential of mitochondrial regulatory networks in TCM modernization. However,interactions among specific bioactive components and clinical translation pathways require further exploration.

##### Synergistic contributions of key monomer components in BYHWD

Studies indicate that Astragalin (AG) and Paeonol (Pae) regulate mitochondrial dynamic balance through distinct molecular mechanisms. In a high-glucose and high-fat-induced HK2 cell injury model, AG significantly inhibits mtROS generation by activating the AMPK/PGC1α pathway while upregulating Mfn2 expression and downregulating Drp1 expression, thereby improving mitochondrial function in the kidneys of STZ-induced diabetic mice [89]. Notably, AG treatment significantly upregulates OPA1, Mfn1, and Mfn2 gene expression and moderately increases mRNA levels of MFF, MiD49, and MiD51 but has no significant effect on Fis1 expression, indicating its specific regulation of mitochondrial fission/fusion balance [90]. In contrast, pae alleviates lipid accumulation in AML12 cells via the AMPK/AKT-Mechanistic target of rapamycin(mTOR)/phosphoinositide 3-kinase(PI3K)/autophagy-related(ATG) pathway axis [91], and in a doxorubicin (Dox)-induced cardiotoxicity model, it selectively upregulates Mfn2 expression to promote mitochondrial fusion through the protein kinase Cepsilon (PKCepsilon)-signal transducer and activator of transcription 3 (Stat3) pathway, without significantly affecting fission proteins such as Drp1 and Fis1 [92]. Comparative analysis reveals that AG more comprehensively regulates mitochondrial biogenesis, while Pae specifically enhances Mfn2-mediated fusion. This complementary mechanism provides new insights for developing combination therapies targeting diabetic complications and chemotherapy-induced toxicity.

#### Qi shen Yi qi dropping pills (QSYQ)

Qi shen Yi qi Dropping Pills(QSYQ), a classical TCM formula, consists of aqueous extracts of *Astragalus membranaceus* (Huang Qi), *Salvia miltiorrhiza Bunge* (Dan Shen), *Panax notoginseng* (Burkill) *F.H.Chen*(San Qi), and *Dalbergia odorifera T.C.Chen* (Huang Tan) [93]. Traditionally used for ischemic [94], myocardial ischemia [95], AS [96], and Alzheimer’s disease(AD) [97], recent studies have revealed QSYQ’s cardioprotective effects through precise modulation of mitochondrial dynamics (fission/fusion balance and biogenesis), providing mechanistic evidence for TCM intervention in energy metabolism disorders. In MI models, QSYQ significantly upregulates mitochondrial fusion proteins OPA1 and Mfn2 while inhibiting phosphorylated Drp1 activity, thereby correcting excessive fission, promoting biogenesis, and ultimately alleviating ischemic myocardial injury [97]. Collectively, QSYQ offers a unique therapeutic strategy for ischemic cardiac injury via mitochondrial dynamic homeostasis. Future studies should elucidate its active components’ regulatory networks on mitochondrial membrane potential, calcium cycling, and ROS metabolism to advance clinical precision.

#### Modified Linggui Zhugan decoction (MLZD)

Linggui Zhugan Decoction (LGZGD), an ancient TCM formula documented in Zhang Zhongjing’s Jin Gui Yao Lue during the Han Dynasty [99], comprises four herbs: *Poria cocos Schw. Wolf* (Fu Ling), *Cinnamomum cassia (L.) J.Presl*(Rou Gui), *Atractylodes macrocephala Koidz.* (Bai Zhu), and *Glycyrrhiza uralensis Fisch.* (Gan Cao) in a 4:3:3:2 ratio [100]. Previous studies primarily applied LGZGD to AD, fatty liver [101], pulmonary hypertension [102], and HF [103]. The modified Linggui Zhugan Decoction (MLZD), derived from LGZGD, includes *Astragalus membranaceus*(Huangqi), *Panax ginseng* (Renshen), *Cinnamomum cassia* (Guizhi), *Poria cocos* (Fuling), *Atractylodes macrocephala* (Baizhu), *Alisma orientale* (Zexie), *Salvia miltiorrhiza* (Danshen), *Areca catechu* (Dafupi), *Lepidium apetalum* (Tinglizi), and *Angelica sinensis* (Danggui). Studies demonstrate its significant cardioprotective effects through multi-target mitochondrial regulation: In MI models, MLZD markedly upregulates Mfn2 expression and suppresses Drp1 phosphorylation(reduced p-Drp1/Drp1 ratio), effectively rectifying mitochondrial dynamic imbalance [104]. In metabolic cardiomyopathy and HF models, LGZGD/MLZD attenuate oxidative injury by inhibiting ferroptosis [105] and activating the SIRT1-AMPK-PGC1αpathway [106], respectively, it is noteworthy that the newly added herbal components in MLZD enhance the regulation of mitochondria through specific mechanisms. Research indicates that Astragaloside IV, the primary active ingredient in Astragalus, suppresses podocyte cell death, increased ROS generation, mitochondrial fragmentation, and dysfunction by inducing the Mfn2/Pink1/Parkin mitophagy pathway both in vivo and in vitro [107]. Complementarily, cinnamaldehyde (CA) from cinnamon twig increases Superoxide dismutase (SOD), M​​atrix ​​M​​etallo​​p​​roteinase (MMP), and Adenosine triphosphate(​ATP) levels, reduces ROS and apoptosis rates, and restores mitochondrial structure; CA also upregulates mRNA expression of Mfn1, Mfn2, Fis1, Drp1, OPA1, and PGC-1α, increases LC3II and p62 expression, and promotes the PINK1/Parkin signaling pathway [108]. Other newly added components in MLZD contribute synergistic effects: Ginsenoside Rb1 (GRb1) significantly improved the reduced Mfn2 expression in cardiac tissues of leptin receptor-deficient db/db mice and in PA-treated cardiomyocytes [109], while Tanshinone IIa reduced mitochondrial fragmentation in MGO-cultured BRECs and increased mRNA levels of Mfn1 and OPA1 [110]. These mechanistic studies demonstrate that MLZD, through its new components, strengthens the regulation of mitochondrial dynamics—particularly with Astragaloside IV primarily enhancing mitophagy via the Mfn2/Pink1/Parkin axis, while Cinnamaldehyde maintains mitochondrial quality by broadly regulating the expression of mitochondrial fusion/fission-related proteins, thereby synergistically enhancing the cardioprotective effects of MLZD.

#### Tongmai Yangxin Pills (TMYX)

Tongmai Yangxin Pills (TMYX), a Chinese patent medicine derived from the traditional formulations "Zhigancao Decoction" and "Shengmai Yin," received approval from the China National Medical Products Administration in 1965 (Approval No.: Z12020589). Its formulation includes 11 herbs such as *Rehmannia glutinosa (Gaertn.) DC*. (Di Huang), *Callerya dielsiana (Harms) P.K. Loc ex Z. Wei & Pedley* (Ji Xueteng), *Ophiopogon japonicus (Thunb.) Ker Gawl.* (Mai Dong), and *Glycyrrhiza uralensis Fisch.* (Gan Cao) [111]. Clinical studies confirm that TMYX improves coronary heart disease [112] and arrhythmia-related Qi-Yin deficiency syndrome [113] via multi-target regulation, with core mechanisms including oxidative stress inhibition [114], n​​uclear ​​f​​actor-​​κ​​appa​​B​​ (NF-κB) inflammatory signaling antagonism [73], and hemodynamic normalization [116]. TMYX’s cardioprotection against MIRI is closely tied to restoring mitochondrial dynamic equilibrium. Transmission electron microscopy (TEM) reveals that high-dose TMYX reverses MIRI-induced mitochondrial swelling, cristae destruction, and vacuolation while dose-dependently improving ultrastructural integrity. Subsequent research indicates that TMYX markedly downregulates the fission proteins Drp1 and Fis1 while upregulating the fusion proteins Mfn1 and Mfn2 [117], while promoting mitochondrial energy metabolism remodeling via the estrogen receptor alpha (ERα)​/PGC-1α axis. Additionally, TMYX enhances coronary microvascular dilation by activating Cyclic Adenosine Monophosphate (​cAMP)/PKA and Nitric Oxide ​(NO)/Cyclic Guanosine Monophosphate (​cGMP) pathways, suppressing the "no-reflow " phenomenon [118]. TMYX integrates multi-component/multi-pathway regulation of inflammation, redox homeostasis, and mitochondrial dynamics, highlighting TCM’s systemic intervention advantages in CVD. Active components (e.g., ophiopogonin, glycyrrhizic acid) may synergistically target energy metabolism nodes, but further studies are needed to clarify component interactions and clinical precision indications for mitochondrial protection-based personalized therapy.

#### Sini decoction (SND)

Sini Decoction (SND), a renowned TCM formula composed of three herbs: *Aconitum carmichaelii Debeaux* (Fu Zi), *Glycyrrhiza uralensis Fisch.* (Gan Cao), and *Zingiber officinale Roscoe* (Gan Jiang) [119], demonstrates anti-inflammatory and microcirculation-improving effects [120, 121]. Its cardioprotective role is widely recognized and officially documented in the Chinese Pharmacopoeia. Clinically, SND is used for HF [122], shock [123], and coronary heart disease to enhance blood circulation in patients with cold limbs, weak pulse, and systemic weakness [124]. SND’s cardioprotection involves mitochondrial energy metabolism and dynamics regulation. 4D proteomics and network pharmacology identify energy metabolism preservation as its primary mechanism [125]. Recent studies reveal that SND’s active components (e.g., songorine, 8-gingerol, isoliquiritigenin) significantly upregulate Mfn1, Mfn2, and OPA1 expression in DOX-induced H9c2 cardiomyocyte toxicity models. The Songorine (SGI) treatment elevates the protein levels of Sdha, Acsl1, Ogdh, Cpt1b, and Cpt2—crucial enzymes in the tricarboxylic acid (TCA) cycle and fatty acid metabolism—thereby alleviating DCM and clarifying SGI's regulatory mechanism on cardiac energy and mitochondrial dysfunction [126].

#### Fufang Zhenzhu Tiaozhi (FTZ)

Fufang Zhenzhu Tiaozhi (FTZ), a herbal compound developed by Prof. Jiao Guo, comprises eight herbs: *Sargentodoxa cuneata (Oliv.) Rehder & E.H.Wilson* (Hong Gen), *Ligustrum lucidum W.T.Aiton* (Nü Zhenzi), *Hypericum japonicum Thunb.* (Tian Jihuang), S*alvia miltiorrhiza Bunge* (Dan Shen), *Panax notoginseng* (San Qi), *Eucommia ulmoides* (Du Zhong), *Citrus reticulata* (Chen Pi), and *Atractylodes macrocephala* (Bai Zhu). FTZ is used for coronary heart disease [127], AS[128], hyperlipidemia, diabetes, non-alcoholic steatohepatitis [129], CH, and hypertension [130]. Previous studies demonstrate FTZ’s broad pharmacological effects: lipid [131] and glucose regulation, anti-inflammation antioxidant activity, insulin resistance alleviation, cardiac lipotoxicity reduction, blood coagulation modulation, and endothelial protection [132, 133]. Recent studies highlight FTZ’s therapeutic role in diabetic cardiomyopathy via mitochondrial dynamics regulation. In high-fat diet/STZ-induced diabetic mice and palmitic acid(PA)-treated H9c2 cardiomyocytes, FTZ suppresses abnormal Drp1/Fis1 upregulation while reversing Mfn2/OPA1 downregulation, restoring mitochondrial fission-fusion balance. Additionally, FTZ improves palmitate-induced mitochondrial respiratory dysfunction by enhancing maximal respiratory capacity (MRC), spare respiratory capacity (SRC), and ATP production, indicating restored oxidative phosphorylation efficiency to mitigate lipotoxic injury [134]. Collectively, FTZ integrates multi-target regulation of lipid metabolism, inflammation, and mitochondrial dynamics, offering a unique formula-based strategy for metabolic CVD. Future research should elucidate how active components (e.g., tanshinones, notoginsenosides) target fission/fusion axes (e.g., Drp1 phosphorylation or OPA1 proteolysis) and develop metabolic phenotype-guided precision therapies.

#### Yiqi Huoxue decoction (YQHX)

Yiqi Huoxue Decoction (YQHX), an herbal formula derived from the classical Danggui Huoxue Decoction, comprises 11 herbs: *Astragalus membranaceus (Fisch.) Bunge* (Huang Qi), *ngelica sinensis (Oliv.) Diels* (Dang Gui), *Prunus persica (L.) Batsch* (Tao Ren), *Carthamus tinctorius L.* (Hong Hua), *Pheretima aspergillum (E. Perrier)* (Di Long), *Paeonia veitchii Lynch* (Chi Shao), *Conioselinum acuminatum (Franch.) Lavrova* (Chuan Xiong), *Angelica dahurica (Hoffm.) Benth. & Hook.f. ex Franch. & Sav.* (Bai Zhi), *Asarum heterotropoides F.Schmidt* (Xi Xin), *Hirudo nipponia Whitman* (Shui Zhi), and *Glycyrrhiza uralensis Fisch.* (Gan Cao) [135]. Clinically used for ischemic heart disease [136], its core active components (e.g., astragaloside IV, calycosin, ferulic acid and ginsenosides Rg1(GRg1)/1GRb1) exert cardioprotection via multi-target synergies: inhibiting myocardial oxidative stress and inflammation, promoting angiogenesis, and maintaining mitochondrial functional homeostasis through lipid metabolism regulation [137]. In aging-related vascular injury models, YQHX ameliorates endothelial progenitor cell (EPC) dysfunction by modulating mitochondrial dynamics. Studies show that YQHX reverses D-galactose (D-gal)-induced mitochondrial hyperfission in senescent EPCs, significantly reducing mRNA and protein levels of fission proteins Drp1, MFF, and Fis1. Further research reveals that YQHX suppresses mitochondrial fission and promotes fusion via AMPK pathway activation, delaying cellular senescence [138]. Notably, AMPK exerts bidirectional regulation of mitochondrial dynamics, with its phosphorylation state dynamically balancing fission and fusion, providing a key molecular target for YQHX’s metabolic-mitochondrial axis intervention.In summary, YQHX highlights TCM’s multi-component, multi-pathway therapeutic advantages through an integrated network of "antioxidant-anti-inflammatory-metabolic regulation-mitochondrial dynamics modulation." Future studies should elucidate the synergistic mechanisms of its active components (e.g., astragaloside IV and ferulic acid) in targeting the AMPK axis and mitochondrial quality control systems (e.g., mitophagy) to advance precision therapies based on metabolic homeostasis regulation (Table 3).

### TCM preparations

#### Shenmai injection (SMI)

Shenmai Injection (SMI), a patented injectable preparation in China, is primarily composed of aqueous extracts from *anax ginseng C.A.Mey.* (Ren Shen) and *Ophiopogon japonicus (Thunb.) Ker Gawl.* (Mai Dong). Developed under TCM theory and modern pharmaceutical technology, SMI is a sterile product extracted and purified from herbal materials [139]. The primary bioactive constituents comprise GRg1, ginsenosides Re(GRe), GRb1, and ophiopogonin D [140]. SMI is widely used to treat CVD [99]. Studies demonstrate that SMI exerts cardioprotection by targeting mitochondrial fusion-fission equilibrium. In hypoxia/reoxygenation (H/R)-induced H9c2 cardiomyocyte injury models, SMI pretreatment significantly upregulates fusion proteins Mfn1, Mfn2, and OPA1 while suppressing mRNA levels of fission proteins Drp1 and Fis1 [142]. Further research reveals that SMI’s active component combination (GRS, containing GRb1, rubusoside, and schisandrin) inhibits Drp1 phosphorylation at Ser616 via AMPK pathway activation, thereby blocking excessive fission, reducing infarct size, and improving cardiac function [143]. Mechanistic studies on Shengmai San extract (ESMS) extend its regulatory role in mitochondrial dynamics: ESMS restores intracellular Ca^2+^ homeostasis, downregulates calcineurin A (CnA) expression, inhibits pro-fission phosphorylation of Drp1 Ser616, and enhances anti-fission phosphorylation at Ser637, dually modulating mitochondrial fission [144]. This effect is validated in oxygen-glucose deprivation (OGD)-injured H9c2 cells, indicating that ESMS suppresses mitochondrial-mediated apoptosis via the "Ca^2+^-CnA-Drp1 phosphorylation " axis, thereby ameliorating myocardial ischemia-induced heart failure.In summary, SMI and its derivatives (GRS, ESMS) synergistically regulate mitochondrial dynamics through AMPK activation, Ca^2+^ homeostasis, and precise modulation of Drp1 phosphorylation sites. This multi-component strategy provides a molecular pharmacological basis for developing novel TCM preparations targeting mitochondrial protection. Future studies should elucidate SMI’s component interactions and mitochondrial-ER crosstalk mechanisms to optimize clinical precision therapies.

##### Synergistic contributions of key monomer components in SMI

The core active components of SMI—GRb1, ginsenoside Rh1 (GRh1), and ginsenoside Rg5 (GRg5)—synergistically regulate mitochondrial dynamic balance through complementary molecular pathways, forming the material basis for its cardiovascular protective effects. GRb1 bidirectionally modulates mitochondrial fission-fusion dynamics by significantly upregulating the expression level of mitochondrial fusion protein Mfn2, restoring the physiological ratio of long/short OPA1 (L-OPA1/S-OPA1), and promoting phosphorylation at the Drp1-Ser637 site, effectively inhibiting excessive mitochondrial fission. In a DCM model, this component successfully reversed ultrastructural damage such as mitochondrial cristae disruption and abnormal lipid droplet accumulation, restoring energy metabolism homeostasis [109, 145]. GRh1 primarily functions via the Sirtuin 3 (​​SIRT3)-dependent signaling pathway, enhancing Mfn2/OPA1-mediated mitochondrial membrane fusion while inhibiting pro-fission phosphorylation at the Drp1-Ser616 site. This regulatory pattern significantly improved mitochondrial network topological integrity in a chronic HF model and alleviated ischemic myocardial injury by coordinating mitophagy with dynamic balance [145–148]. The molecular action of GRg5 focuses on regulating Drp1 activity by activating the AKT signaling pathway to promote phosphorylation at the Drp1-Ser637 site, stabilizing it in an inactive conformational state. This mechanism effectively blocks the mitochondrial fission cascade, inhibits reactive oxygen species bursts and calcium overload, rapidly restores mitochondrial membrane potential, and enhances cell survival rates [148–151]. Notably, these three ginsenosides form a temporal synergistic effect through the AMPK-Ca^2^⁺-Drp1 phosphorylation signaling axis: GRb1 establishes the foundation for mitochondrial dynamic balance, GRh1 enhances structural stability, and GRg5 provides rapid intervention for acute injuries, collectively constructing the core pharmacological framework of SMI's "multi-component, multi-target" regulation of mitochondrial dysfunction. Current research has not fully elucidated GRg5's regulatory potential on fusion proteins, and its pharmacokinetic characteristics within the endoplasmic reticulum-mitochondria interaction network require further exploration.

#### Danqi soft capsules (DQ)

Danqi Soft Capsules (DQ), a TCM preparation composed of *Salvia miltiorrhiza Bunge* (Dan Shen) and *Panax notoginseng* (San Qi), is clinically used for cardiovascular and cerebrovascular diseases. Salvianolic acids (derived from Danshen) and notoginsenosides (extracted from Sanqi) are recognized for their ability to mitigate cardiomyocyte apoptosis through mitochondrial mechanisms [152]. In MIRI rat models, DQ pretreatment significantly alleviates mitochondrial ultrastructural disarray: electron microscopy reveals that DQ restores regular myofilament alignment and cristae morphology while increasing intact mitochondrial numbers in both low- and high-dose groups. Mechanistically, DQ inhibits Drp1 phosphorylation and aberrant Ca^2+^/calmodulin-dependent protein kinase II (p-CaMKII) activation, blocking pathological mitochondrial fission [153]. This protective effect is further confirmed in H₂O₂-induced cardiomyocyte injury models, demonstrating DQ’s cytoprotection via reduced mitochondrial fragmentation (Table 3).

### TCM crude extracts

#### Lycium barbarum polysaccharide (LBP)

Lycium barbarum polysaccharide (LBP), a cardioprotective TCM extract, ameliorates MIRI by regulating mitochondrial fission and fusion dynamics. In H_2_O_2_-induced H9c2 cell ischemia/reperfusion (I/R) models, LBP rectifies mitochondrial dynamic imbalance by downregulating Drp1 and upregulating OPA1/Mfn2. The cardioprotective mechanism of LBP involves G protein-coupled receptor kinase 2 (GRK2) signaling: in cells with GRK2 overexpression, the mitochondrial regulatory effects of LBP are reduced, whereas GRK2 inhibitors replicate LBP's actions, establishing GRK2 as a critical target. Furthermore, LBP reinstates the activity of the AKT/eNOS pathway, thereby suppressing I/R-induced apoptosis and oxidative stress. This study introduces a unique "GRK2-mitochondrial dynamics" axis for the multi-pathway protection of LBP, offering experimental proof for its CVD applications and insights into TCM extracts that target energy metabolism [154].

#### Astragalus radix (AR) combined with Notoginseng radix et Rhizoma (NR)

Astragalus Radix (AR) and Notoginseng Radix et Rhizoma (NR), a classic TCM pair (abbreviated as AN), significantly improves cardiac dysfunction and pathological remodeling in transverse aortic constriction (TAC)-induced mice. AN bidirectionally regulates mitochondrial dynamics: in TAC models, AN suppresses p-Drp1 Ser616 while upregulating Mfn2 and OPA1, restoring fusion-fission balance. In vitro studies confirm AN’s consistent regulation of Drp1/Mfn2/OPA1, highlighting its core mechanism of maintaining mitochondrial dynamic homeostasis to counteract myocardial injury [155].

### Broad-spectrum active monomers

#### Baicalin (BA)

Baicalin (BA), a flavonoid derived from the roots of Scutellaria baicalensis, demonstrates anti-inflammatory and antioxidant characteristics [151] and demonstrates therapeutic potential in CVD [156]. Research confirms its protective role in post-cardiac arrest (CA) myocardial injury by targeting mitochondrial fission protein Drp1. In asphyxia-induced CA rat models, BA treatment for 4 weeks significantly improves post-resuscitation survival rates, accompanied by reduced p-Drp1 Ser616 levels, inhibited Drp1 mitochondrial translocation, and diminished mitochondrial fission. Further validation in H9c2 cell I/R models shows that BA (20 μmol/L) and the Drp1-specific inhibitor Mdivi-1 both suppress p-Drp1 Ser616 and fission, reduce ROS generation, cytochrome c release, and cardiomyocyte death, and enhance mitochondrial respiratory function. Notably, although mitochondrial fission receptors MiD49 and Fis1 are upregulated in CA rats, BA does not significantly affect their expression, indicating its focus on Drp1 activity regulation rather than receptor synthesis. Additionally, BA’s protective effects may involve AMPK signaling pathway activation. This study elucidates BA’s molecular mechanism in ameliorating MIRI via the "Drp1 phosphorylation inhibition-mitochondrial fission blockade" axis [157].

#### Salidroside (Sal)

Salidroside (Sal), the primary active component of Rhodiola rosea, exhibits anti-inflammatory, antioxidant, and mitochondrial protective. Its therapeutic potential in CVD has been validated across multiple models [157–161]. In MI and I/R injury, Sal significantly mitigates mitochondrial swelling and cristae degradation, augments mtDNA expression and ATP levels, and reinstates mitochondrial network integrity by upregulating macrophage migration inhibitory factor (MIF) to activate AMPK phosphorylation, thereby enhancing the expression of fusion proteins OPA1 and Mfn1—effects that are more pronounced at elevated concentrations [162]. Electron microscopy reveals that I/R injury induces mitochondrial fragmentation (reduced mean area and increased mitochondrial number/μmol/L^2^, *P* < 0.0001), which Sal intervention significantly reverses (*P* < 0.0001). Sal also suppresses pro-fission factor Fis1 expression (*P* = 0.0003) and p-Drp1 Ser616 phosphorylation (*P* = 0.0001), with its effects dependent on the AMPK pathway (AMPK inhibitor compound C completely blocks Sal’s effects, *P* < 0.01). Further studies demonstrate that Sal inhibits mitochondrial fission via the AMPK-Drp1 axis [163]. In high glucose-induced vascular smooth muscle cells (VSMCs), Sal downregulates Drp1, upregulates Mfn2 (significantly increasing filamentous mitochondria proportion, *P* < 0.05), and reduces ​​Nicotinamide Adenine Dinucleotide Phosphate (NADPH) oxidase activity to lower ROS levels. Mfn2 silencing partially reverses Sal’s antioxidant effects (NADPH oxidase activity difference *P* < 0.05), indicating that Sal upregulates Mfn2 to suppress ROS partly by inhibiting NADPH oxidase [164]. Collectively, Sal integrates the "MIF/AMPK signaling axis" to synergistically balance fission-fusion dynamics (inhibiting Drp1 and promoting OPA1/Mfn2) while coordinating antioxidant and metabolic pathways, offering a multi-target strategy for myocardial injury and metabolic disorder-related CVD.

#### Curculigoside E (CE)

Curculigoside E (CE), a natural pentacyclic triterpenoid saponin and main active component of Aralia elata, exerts anti-CVD effects [165, 166]. Studies show that CE protects against I/R injury by coordinately regulating mitochondrial fission-fusion balance and calcium homeostasis. TEM demonstrates that CE significantly improves mitochondrial ultrastructural disarray in cardiomyocytes, suppresses Drp1 expression, and restores fusion proteins Mfn1/2 and OPA1 levels. Mechanistically, CE activates the AMPK pathway to upregulate p-AMPK and OPA1 expression, promoting mitochondrial fusion and alleviating I/R-induced mitochondrial dysfunction [167]. These findings provide experimental evidence for CE’ therapeutic strategy targeting mitochondrial dynamics in myocardial I/R injury.

#### Breviscapine (BRE)

Breviscapine (BRE), a flavonoid derived from Erigeron breviscapus, demonstrates antioxidant, anti-inflammatory, and cardiovascular protective effects [168]. In phenylephrine (PE)-induced cardiomyocyte hypertrophy and TAC-induced HF models, BRE targets mitochondrial fusion protein Mfn1 to regulate dynamics. PE treatment significantly increases mtROS accumulation and downregulates Mfn1 expression, while BRE pretreatment dose-dependently inhibits ROS bursts and reverses Mfn1 suppression caused by PE or TAC, without affecting Mfn2 levels. Further research reveals that BRE activates forkhead box O3a (FOXO3a) transcription factor to enhance Mfn1-mediated mitochondrial fusion, thereby improving mitochondrial morphology/function and alleviating CH and HF [169].

#### Berberine (BBR)

Berberine (BBR), the primary active component of the TCM Coptis chinensis (Huanglian), exhibits anti-inflammatory and cardiovascular protective effects. It is extensively utilized in the management of cardiovascular disorders including AS, CH, and HF [170]. Research indicates that in high glucose-induced cardiac injury, BBR mitigates excessive mitochondrial fission by limiting the mitochondrial translocation of Drp1 protein and downregulating its expression, therefore alleviating hyperglycemia-induced cardiomyocyte damage [171]. In a "double-hit" diet-induced HF model, BBR restores autophagy flux, alleviates Drp1-mediated mitochondrial fragmentation and calcium overload, and promotes phosphorylation of the sarcoplasmic reticulum calcium pump SERCA2a, accelerating cytosolic calcium reuptake into the sarcoplasmic reticulum and subsequently improving cardiac diastolic dysfunction [172]. Collectively, BBR provides a multi-target intervention strategy for hyperglycemic myocardial injury and heart failure by targeting Drp1 to inhibit pathological mitochondrial fission, restoring calcium homeostasis, and enhancing autophagy function.

#### Resveratrol (RSV)

Resveratrol (RSV), a natural polyphenolic substance sourced from Polygonum cuspidatum (Huzhang), exhibits antioxidant, anti-inflammatory, and cardioprotective effects [173, 174]. Studies indicate that in MIRI models, RSV activates the Sirt1/Sirt3-Mfn2-Parkin-PGC-1α signaling pathway, markedly enhancing mRNA expression of Fis1, Mfn1, Mfn2, Drp1, and OPA1, thus facilitating mitochondrial fission-fusion homeostasis and biogenesis. This reinstates mitochondrial quality control and alleviates cardiac damage [175]. In D-galactose-induced senescent-like cardiomyocytes, RSV dose-dependently suppresses excessive mitochondrial elongation by upregulating Drp1 expression and activating the Parkin/PINK1 pathway, reversing mitochondrial morphological abnormalities and functional dysregulation [176]. Additionally, in PA-induced endothelial oxidative stress models, RSV upregulates fusion proteins OPA1, Mfn1, and Mfn2, promoting mitochondrial tubular structure formation and reducing fragmentation and oxidative damage via the TyrRS-​PARP1 signaling pathway [174]. These findings collectively reveal that RSV improves mitochondrial morphology, function, and oxidative stress responses through dynamic regulation of mitochondrial dynamics, offering a multi-level mitochondrial-targeted intervention strategy for AS and ischemic heart disease (Table 4,1,2,3).

**Table 1:** Mitochondrial Dynamics-Related Proteins

Protein category	Protein name	Mechanism of action	References
Mitochondrial Fission-Related	Drp1	Core fission protein mediating mitochondrial membrane scission via GTP-dependent oligomerization; regulated by diverse post-translational modifications including phosphorylation (S616 promotes, S637 inhibits), SUMOylation, S-nitrosylation, ISGylation, O-GlcNAcylation, and K642 acetylation.	[6, 15, 19–21, 25–29]
	Fis1	Mitochondrial outer membrane receptors bind to the GTPase effector domain of Drp1 and recruit Drp1 to mitochondrial fission sites.	[17, 18]
	MFF	The C-terminal transmembrane domain anchors to the OMM, while the N-terminal R1-R2 motif and coiled-coil domain directly bind Drp1, facilitating Drp1 translocation to mitochondria and initiating mitochondrial fission.	[17, 18]
	MiD49/MiD51	Facilitates Drp1 recruitment to mitochondria and cooperates with MFF to regulate fission dynamics.	[17, 18]
Mitochondrial Fusion-Related	Mfn1/Mfn2	Mediates mitochondrial outer membrane fusion	[16, 20, 31]
	OPA1	Mediates mitochondrial inner membrane fusion	[16, 32]

**Table 2:** Mitochondrial Fusion and Fission-Related Pathways

Pathway classification	Pathway name	Mechanism of action	Key molecules	Regulatory direction	References
Mitochondrial Fission Signaling Pathway	PKA signaling pathway	AKAP1 anchors PKA to mitochondria, promoting Drp1 phosphorylation at Ser637/Ser656	PKA, AKAP1, Drp1	Pro-Fission	[47–50]
	Drp1/Fis1 signaling pathway	Fis1 binds to the Drp1-GED (GTPase effector domain), recruiting Drp1 to fission sites and promoting mitochondrial fission.	Drp1, Fis1, MiD49/51	Pro-Fission	[17, 18]
	MFF/Drp1 signaling pathway	MFF functions as the primary fission receptor, facilitating Drp1 translocation to mitochondria and initiating mitochondrial fission. Phosphorylation of MFF enhances Drp1 recruitment to mitochondria.	MFF, Drp1, AMPK,	Pro-Fission	[35, 36]
Mitochondrial membrane fusion signaling pathway	AMPK/Drp1signaling pathway	Under energy stress, AMPK activates MFF phosphorylation, thereby promoting Drp1 recruitment to mitochondria.	AMPK, Drp1, MFF	Bidirectional Regulation	[36–40] [60]
	AMPK signaling pathway	AMPK activation promotes mitochondrial elongation/fusion by stabilizing Mfn1/2 and OPA1 (inhibiting their degradation) while suppressing the upregulation of Drp1.	AMPK,Drp1(please delete the Drp1), Mfn2, OPA1	Bidirectional Regulation	[40–43]
	SIRT1 signaling pathway	SIRT1 activation regulates PGC-1α to suppress Drp1/Fis1 expression while upregulating OPA1.	SIRT1, PGC-1α, Drp1,Fis1, OPA1	Pro-Fusion	[43–47]

**Table 3:** Therapeutic Targeting of Mitochondrial Dynamics by TCM in CVD (all of the plant name had been checked with http://www.theplantlist.org/)

TCM Name	Composition	Mechanism of Action	Experimental Indicators	Experimental Model	References
Buyang Huanwu Decoction	*Astragalus membranaceus*, *Angelica sinensis (Oliv.) Diels*, *Paeonia lactiflora Pall.*, *Conioselinum acuminatum (Franch.) Lavrova*, *Prunus persica (L.) Batsch*, *Carthamus tinctorius L.and Pheretima aspergillum (E. Perrier)*	Targets the AMPK-Drp1 signaling axis to significantly suppress excessive mitochondrial fission	Reduced myocardial mitochondrial fission, improved endothelial function	STZ-induced diabetic mice	[81–88]
Qishen Yiqi Dropping Pills	*Aqueous extracts of Astragalus membranaceus*, *Salvia miltiorrhiza Bunge*, *Panax notoginseng (Burkill) F.H.Chen*, and *Dalbergia odorifera T.C.Chen*	Upregulates OPA1/Mfn2 expression and inhibits p-Drp1	Correct excessive fission and alleviate ischaemic myocardial injury.	MI model	[92–55]
Modified Linggui Zhugan Decoction	*Poria cocos Schw. Wolf*, *Cinnamomum cassia (L.) J.Presl*, *Atractylodes macrocephala Koidz.*, and* Glycyrrhiza uralensis Fisch.*	Upregulates Mfn2 expression and inhibits Drp1 phosphorylation while activates the SIRT1-AMPK-PGC1α pathway	Improved cardiac function	Metabolic Cardiomyopathy Rat Model, HF Mouse Model	[98–110]
Tongmai Yangxin Pill	*Rehmannia glutinosa (Gaertn.) DC.*, *Callerya dielsiana (Harms) P.K. Loc ex Z. Wei & Pedley*, *Ophiopogon japonicus (Thunb.) Ker Gawl.*and *Glycyrrhiza uralensis Fisch.*	Downregulates Drp1/Fis1 expression, upregulates Mfn1/2 expression, and activates the ERα/PGC-1α signaling axis	Cardiac function ↑, Apoptosis ↓,	MIRI Rat Model and H/R-Injured H9c2 Cell Model	[110–113,], [114–118-76]
Sini Decoction	*Aconitum carmichaelii Debeaux*, *Glycyrrhiza uralensis Fisch.* and *Zingiber officinale Roscoe*	Upregulates Mfn1/2 and OPA1 expression, promoting the expression of TCA cycle-related enzymes	ATP production ↑, mitochondrial respiratory function restored	DOX-Induced H9c2 Cardiomyocyte Model (In Vitro)	[118–125]
Fufang Zhenzhu Tiaozhi	Sargentodoxa cuneata (Oliv.) Rehder & E.H.Wilson, *Ligustrum lucidum W.T.Aiton*, *Hypericum japonicum Thunb.*, *Salvia miltiorrhiza Bunge*, *Panax notoginseng*, *Eucommia ulmoides*, *Citrus reticulata* and *Atractylodes macrocephala*	Inhibits Drp1/Fis1, upregulates Mfn2/OPA1, and improves mitochondrial respiratory function	Mitochondrial respiratory capacity ↑, lipid droplet accumulation ↓	High-fat diet combined with STZ-induced diabetic mouse model, PA-treated H9c2 cardiomyocytes	[126–134]
Yiqi Huoxue Decoction	*Astragalus membranaceus (Fisch.) Bunge*, *ngelica sinensis (Oliv.) Diels*, *Prunus persica (L.) Batsch*, *Carthamus tinctorius L.*, *Pheretima aspergillum (E. Perrier)*, *Paeonia veitchii Lynch*, *Conioselinum acuminatum (Franch.) Lavrova*, *Angelica dahurica (Hoffm.) Benth. & Hook.f. ex Franch. & Sav.*, *Asarum heterotropoides F.Schmidt*, *Hirudo nipponia Whitman*, and *Glycyrrhiza uralensis Fisch.*	Activates AMPK pathway, inhibits Drp1/MFF/Fis1 expression	Mitochondrial fission reduced, senescence-associated markers ↓	D-gal-induced senescent EPCs model	[134–138]
Shenmai Injection	Aqueous Extracts of *anax ginseng C.A.Mey.* and *Ophiopogon japonicus (Thunb.) Ker Gawl.* Roots	Activates the AMPK pathway, upregulates Mfn1/2 and OPA1 expression, inhibits Drp1 and Fis1 mRNA expression, suppresses p-Drp1 Ser616, and promotes p-Drp1 Ser637	Myocardial infarct size ↓, mitochondrial fission reduced	H/R- and OGD-Induced H9c2 Cardiomyocyte Injury Models	[138–144]
Danqi Soft Capsule	*Salvia miltiorrhiza Bunge*and *Panax notoginseng*	Inhibition of Drp1 phosphorylation and CaMKII activation	Mitochondrial structural integrity restored, apoptosis rate ↓	MIRI Rat Model and H_2_O_2_-Induced Cardiomyocyte Injury Model	[152, 153]

#### Vitexin (VT)

Vitexin (VT), a flavonoid derived from Vitex and Crataegus (Hawthorn) plants, exhibits anti-vascular inflammatory and cardiovascular protective activities [176, 178] Research indicates that VT mitigates MIRI by modulating mitochondrial fission/fusion balance. In rat MIRI models, VT, either alone or in conjunction with the Epac inhibitor ESI-09, markedly enhances mitochondrial structural integrity, evidenced by restored outer membranes and cristae, while inhibiting the aberrant overexpression of the fission protein Drp1 and upregulating the fusion protein Mfn2. VT mechanistically activates the Epac1-Rap1 signaling pathway to rectify the I/R-induced imbalance in mitochondrial fission and fusion, diminishing Drp1-mediated pathological fission and augmenting Mfn2-dependent fusion capability. Moreover, VT collaborates with ESI-09 to enhance cardioprotection, while its intervention markedly mitigates the adverse effects of the pro-injury agent 8-CPT [179].

#### Gastrodin (GAS)

Gastrodin (GAS), the principal active constituent of the TCM *Gastrodia elata* (Tianma), demonstrates neuroprotective and cardiovascular protective properties [180, 181]. Studies demonstrate that in H_2_O_2_-treated H9c2 cardiomyocytes, GAS pretreatment significantly inhibits mitochondrial fragmentation, reverses H_2_O_2_-induced downregulation of fusion proteins Mfn2 and OPA1, and suppresses fission protein Fis1 upregulation, while showing no significant effect on Mfn1 or Drp1 expression. Further research reveals that GAS improves mitochondrial respiratory function, increases tubular length and network complexity by restoring Mfn2/OPA1-mediated fusion capacity and inhibiting Fis1-dependent fission, thereby alleviating oxidative stress-induced mitochondrial membrane potential collapse and energy metabolism dysfunction [182].

#### Cordycepin (COR)

**Fig. 2:**
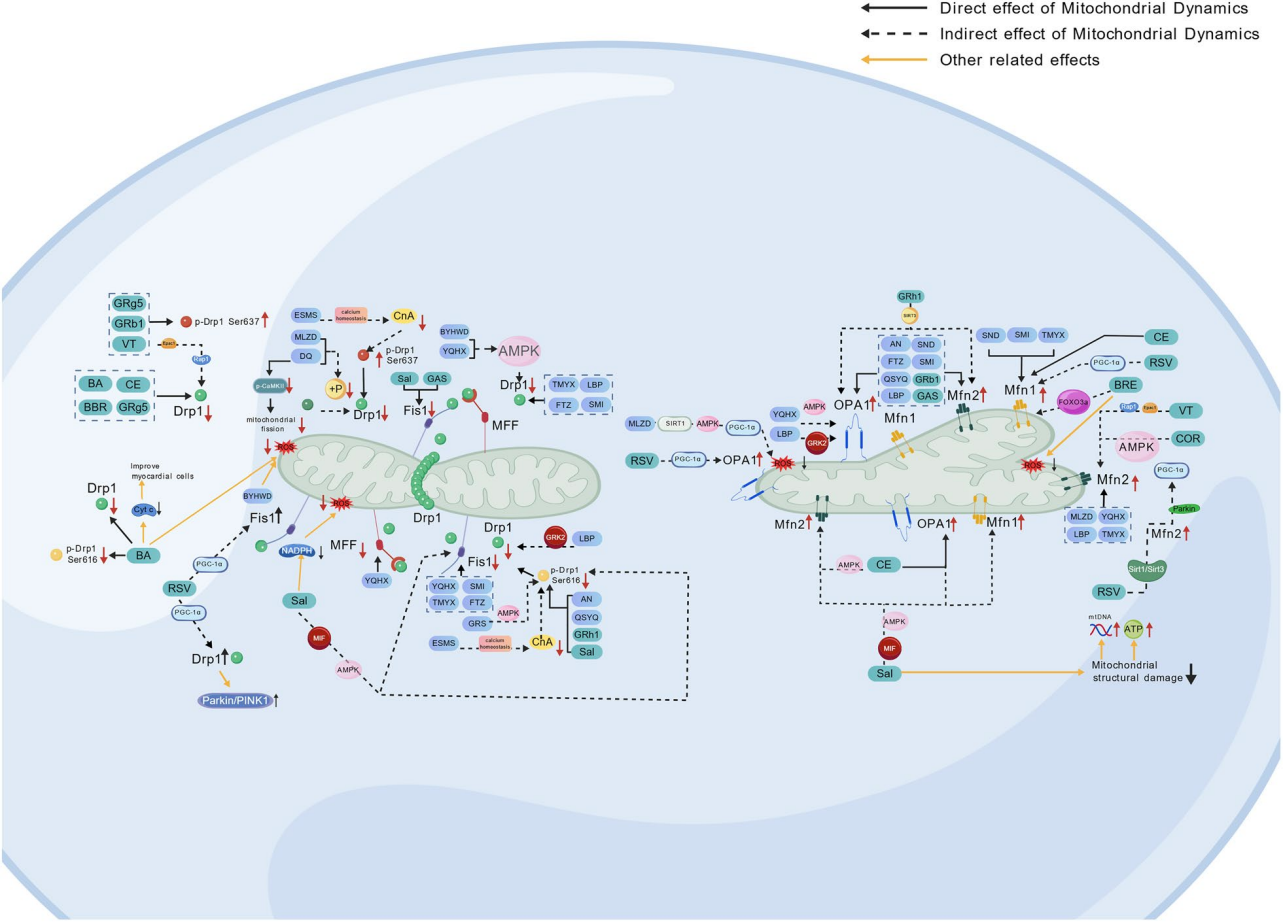
​​ Schematic Diagram of the Mechanism by Which TCM Intervenes in CVD Through Regulation of Mitochondrial Dynamics​.This study thoroughly clarifies how TCM medic compound formulae, preparations, crude extracts, and their active monomers collaboratively influence mitochondrial dynamics through multi-target and multi-pathway mechanisms. Representative TCM interventions, including compound formulas like BYHWD and SMI, preparations such as DQ, and crude extracts like the LBP and AN, precisely regulate critical targets of mitochondrial fission and fusion, including Drp1, Mfn2, and OPA1, along with their associated signaling pathways, such as AMPK and Ca²⁺/calcineurin, via their principal active monomers, including astragaloside IV and GRb1. This modulation mitigates pathological processes like oxidative stress, calcium overload, and apoptosis, ultimately providing cardiovascular protection

Cordycepin (COR), an active molecule derived from *Cordyceps sinensis* (Dongchongxiacao), demonstrates many pharmacological activities, including anti-inflammatory, antioxidant [183]. In diabetic mice with combined MIRI, TEM analysis shows that COR significantly suppresses excessive mitochondrial fragmentation, upregulates Mfn2 expression, and improves mitochondrial respiratory function and energy metabolism homeostasis. Mechanistic studies reveal that COR enhances mitochondrial fusion capacity by activating the AMPK signaling pathway to promote Mfn2 transcription and expression. Cardiac-specific Mfn2 knockout experiments confirm that Mfn2 deficiency completely abolishes COR’s cardioprotective effects, indicating its high dependency on Mfn2-mediated mitochondrial dynamic equilibrium [33] in Fig. 2 and Table 4.

**Table 4:** Mitochondrial Dynamics-Targeting Monomer Components from Traditional Chinese Medicine for CVD Regulation

Active monomer components	Mechanism of action	Experimental indicators	Experimental model	Chemical formula	Chemical structure	References
Baicalin	Inhibits p-Drp1 Ser616 to reduce mitochondrial fission	Cell viability↑, cytochrome c release↓	Asphyxia-Induced Cardiac Arrest Rat Model, H9c2 Cell I/R Model	C_21_H_18_O_11_		[151, 156, 157]
Salidroside	Activation of the MIF/AMPK pathway inhibits Drp1 mitochondrial translocation	Drp1 Mitochondrial Translocation↓, ATP Production↑	MI and I/R Injury Models	C_14_H_20_O_7_		[157–163]
Curculigoside E	Activation of the AMPK signaling pathway enhances OPA1-dependent mitochondrial fusion	Mitochondrial Dysfunction Amelioration	I/R Injury Models	C_36_H_56_O_9_		[164–167]
Breviscapine	Activation of FOXO3a transcription factor promotes Mfn1-mediated mitochondrial fusion	Improvement of Mitochondrial Morphology and Function	I/R Injury Models	C_21_H_18_O_12_		[168, 169]
Berberine	Inhibition of Drp1 mitochondrial translocation and downregulation of its expression	Improvement of Cardiomyocyte Injury	HG-Induced Myocardial Injury Model, "Double-Hit" Diet-Induced HF Model	C_20_H_18_NO_4_		[169–172]
Resveratrol	Activates the Sirt1/Sirt3-Mfn2-Parkin-PGC-1α signaling axis to promote mitochondrial fission-fusion balance and biogenesis	Restoration of mitochondrial quality control	MIRI Model, D-gal-induced Senescence-like Cardiomyocyte Model, PA-induced Endothelial Oxidative Stress Model	C_14_H_12_O_3_		[172–176]
Vitexin	Activates Epac1-Rap1 pathway to reverse fission/fusion imbalance	Enhanced cardioprotective effects	MIRI Model	C_21_H_20_O_10_		[176–179]
Gastrodin	Restores Mfn2/OPA1-mediated mitochondrial fusion capacity	Improved mitochondrial respiratory function	H_2_O_2_-treated H9c2 Cardiomyocyte Model	C_13_H_18_O_7_		[179–18181]
Cordycepin	Activates AMPK pathway to promote Mfn2 transcription/expression	Enhanced mitochondrial fusion capacity	Diabetic Mouse Model with MIRI	C_10_H_13_N_5_O_3_		[183, 33]

## Conclusions and perspectives

We present the TCM-Mitochondrial Dynamics Intervention Model (TCM-MDIM), which combines organelle-level regulation with Qi-Blood harmonization. This concept posits that "Yin-Yang imbalance" leads to mitochondrial fission/fusion dyshomeostasis, while multi-component TCM bidirectionally regulates the Drp1-Mfn2-OPA1 axis.

Through fundamental mechanisms including AMPK/SIRT1 regulation, TCM formulations (like BYHWD) and active substances (like Sal) balance mitochondrial dynamics and reduce cardiovascular damage. Although component interactions and bioavailability issues need more research, their combined effect offers novel insights for medication development.

This work illustrates TCM's unique capacity to simultaneously target fission/fusion proteins, creating Yin-Yang dynamic equilibrium, by constructing a 'compound formula-organelle-function' paradigm (Fig. 2). In cardiovascular therapy, the TCM-MDIM offers a scientific basis for evidence-based precision medicine.

## Data Availability

No data was used for the research described in the article.

## Abbreviations

AKAP1: A-kinase anchoring protein 1
AMPK: AMP-activated protein kinase
AR: Astragalus Radix
AS: Atherosclerosis
BA: Baicalin
BBR: Berberine
BRE: Breviscapine
BYHWD: Buyang Huanwu Decoction
CDK1: Cyclin-dependent kinase 1
CE: Curculigoside E
CH: Cardiac hypertrophy
COR: Cordycepin
CVD: Cardiovascular diseases
DCM: Diabetic cardiomyopathy
DQ: Danqi Soft Capsules
Drp1: Dynamin-related protein 1
Fis1: Mitochondrial fission 1 protein
FTZ: Fufang Zhenzhu Tiaozhi
GAS: Gastrodin
GRb1: Ginsenoside Rb1
GRg5: Ginsenoside Rg5
GRh1: Ginsenoside Rh1
GRK2: G protein-coupled receptor kinase 2
I/R: Ischemia/Reperfusion
IMM: Inner mitochondrial membrane
IRI: Myocardial ischemia-reperfusion injury
LBP: Lycium Barbarum Polysaccharide
L-OPA1: Long-form optic atrophy 1
MAMs: Mitochondria-associated membranes
MFF: Mitochondrial fission factor
Mfn1/2: Mitofusin 1/2
MI: Myocardial infarction
MLZD: Modified Linggui Zhugan Decoction
mPTP: Mitochondrial permeability transition pore
mTOR: Mechanistic target of rapamycin
NLRP3: NOD-like receptor family pyrin domain containing 3
OMM: Outer mitochondrial membrane
OPA1: Optic atrophy 1
Pae: Paeonol
PGC-1α: Peroxisome proliferator-activated receptor gamma coactivator 1-alpha
PKA: Protein kinase A
QSYQ: Qishen Yiqi Dropping Pills
ROS: Reactive oxygen species
RSV: Resveratrol
Sal: Salidroside
S-OPA1: Short-form optic atrophy 1
SIRT1: Sirtuin 1
SMI: Shenmai Injection
SND: Sini Decoction
TMYX: Tongmai Yangxin Pills
TCM: Traditional Chinese Medicine
VT: Vitexin
Wnt: Wingless-type MMTV integration site family
YQHX: Yiqi Huoxue Decoction
